# Prevalence and Characteristics of Enteric Pathogens Detected in Diarrhoeic and Non-Diarrhoeic Foals in Trinidad

**DOI:** 10.1155/2012/724959

**Published:** 2012-06-21

**Authors:** Robin Harris, Kerri Sankar, Julie-Anne Small, Rod Suepaul, Alva Stewart-Johnson, Abiodun Adesiyun

**Affiliations:** School of Veterinary Medicine, Faculty of Medical Sciences, University of the West Indies, St. Augustine, Trinidad and Tobago

## Abstract

The study determined the relative importance of *Escherichia coli, E. coli* O157, *Salmonella* spp., *Clostridium* spp., rotavirus, *Cryptosporidium* spp., and *Strongyloides westeri* in foal (diarrhoeic and non-diarrhoeic) available for sampling during the foaling season of 2010 and determined their sensitivity to antimicrobial agents. A cross-sectional study was conducted on 164 foals (9 diarrhoeic and 155 non-diarrhoeic) from 15 farms in Trinidad. Isolation and detection of enteric pathogens followed standard methods, and the antibiograms of *E. coli* and *Salmonella* spp. were determined using the disc diffusion method. All organisms investigated were detected except *E. coli* O157. A high prevalence of *E. coli* (85.0%), *Cryptosporidium* spp. (64.8%), *Strongyloides westeri* (35.7%) was seen, but the prevalence was comparatively low for *Clostridium* spp. (12.9%), *Salmonella* spp. (4.4%) and rotavirus (2.1%). Only *Salmonella* spp. was isolated at a statistically significantly (*P* < 0.05; *χ* ^2^) higher frequency from diarrhoeic (25.0%) than non-diarrhoeic (4.0%) foals. Amongst *E. coli* isolates, the frequency of resistance was higher in isolates from diarrhoeic compared with non-diarrhoeic foals but the difference was only statistically significant (*P* < 0.05; *χ* ^2^) for tetracycline. All isolates of *Salmonella* spp. were sensitive to streptomycin and sulphamethoxazole/trimethoprim, a finding that may have therapeutic significance.

## 1. Introduction

Worldwide, foals have been demonstrated to experience diarrhoeic episodes with resulting mortalities and economic consequences resulting from the morbidities, cost of treatment, and mortalities [1, 2]. It has been reported that up to 80% of foals would have at least one episode of diarrhoea within the first six months of their life resulting in severe dehydration or secondary infection in convalescents [3, 4]. Various agents, including bacteria, parasites, and viruses, have been implicated in morbidities and mortalities encountered by foals [3–6]. It has also been established that apparently healthy, non-diarrhoeic foals are also carriers of these enteric pathogens [5–7].

 The frequency of occurrence of diarrhoea in foals, as well as detected of enteric pathogens have been documented to be affected by host factors including age and sex, management practices, and locations of horse farms amongst other factors [2, 3, 6].

*Escherichia coli* (*E. coli*), despite being part of the normal flora of the gastrointestinal tract of foals and other animals [8], is also known to be an important aetiological agent of foal diarrhoea [9]. The presence of virulence markers in infecting strains of *E. coli* has been demonstrated to be responsible for the pathogenicity detected in infected foals [9, 10].

*Salmonella *spp. have been reported to be frequently isolated from foals [4, 5, 11, 12] and have been identified as causative agents for diarrhoea episodes and epidemics [13, 14]. Serotypes of *Salmonella *including Ohio and Typhimurium have been recovered from diarrhoeic foals [13, 15].

*Clostridium *spp., particularly *C. difficile* and *C. perfringens,* have been documented as aetiological agents for diarrhoea in foals [6, 16]. In a study on the frequency of detection of pathogens in foals with diarrhoea, *C. perfringens* was reported as the second most frequently detected pathogen (18%) after rotavirus (20%) [5]. 

 Rotavirus is considered an important aetiological agent for foal diarrhoea and high mortality rates in foals [2–5, 17]. Reported rates of detection or isolation of rotavirus in diarrhoeic foals have varied between countries [18–20].

*Cryptosporidium parvum* (*C. parvum)* is another pathogen that has been implicated as a causative agent of foal diarrhoea [4–6, 21, 22] but it has also been detected in non-diarrhoeic foals as documented by Burton et al. [23] who found that the frequency of detection of the pathogen was not statistically significantly different for diarrhoeic and non-diarrhoeic foals. Frequency of detection of *C. parvum* has been reported to vary between countries and the risk factors associated with cryptosporidiosis have been described [24–26]. 

 Endoparasites, including *Strongyloides westeri*, strongyles, ascarids, and *Eimeria* spp., have been detected in foals and have also been implicated as causative agents for diarrhoea in foals [4, 5, 25]. It has however been reported that a number of endoparasites are detected in foals exhibiting diarrhoea, as well as in apparently healthy foals [27, 28]. Different rates of detection of endoparasites were reported to vary across stud farms as conveyed by Lyons and Tolliver [29], who stated that frequency of detection of *S. westeri *was 1.5% compared to 27.6% for strongyles in Kentucky. 

 In Trinidad and Tobago, it is estimated that approximately 200 foals are born every year (Trinidad and Tobago Racing Authority, TTRA, personnel, unpublished). In the country, there are a small number of stud farms which produce Thoroughbred foals for the local racing industry. Although local equine practitioners have indicated that foals on local farms experience a high incidence of diarrhoea annually with resulting mortalities, there is a dearth of information on the aetiological agents responsible for episodes of foal diarrhoea in the country. Local practising equine practitioners have reported that, despite early interventions by the use of antimicrobial agents, the success rate was low. 

 In the country, bacterial, parasitic, and viral enteropathogens including rotavirus, *Salmonella *spp., *Cryptosporidium* spp., enteropathogenic and verocytotoxigenic *E. coli*, *Campylobacter* spp., *Yersinia enterocolitica*, and endoparasites have all been documented in young livestock (calves, lambs, and piglets) [30].

 This cross-sectional study was therefore conducted to determine the frequency of detection of selected pathogens, specifically, rotavirus,* Salmonella* spp., *Cryptosporidium *spp., *Clostridium* spp., *Escherichia coli* including *E. coli *O157 and *Strongyloides westeri* in both diarrhoeic and non-diarrhoeic foals. Another objective was to relate the occurrence of enteric pathogens to the foals sampled (sex, age, health history) and farm characteristics (practice, size, location etc). Finally, to determine the sensitivity of isolates of *E. coli* and *Salmonella *spp. to commonly used antimicrobial agents in the equine industry in Trinidad. 

## 2. Materials and Methods

### 2.1. Study Design

 This was a cross-sectional study which entailed visits to selected horse farms during which samples were collected from diarrhoeic and non-diarrhoeic foals. Each farm and selected foal were sampled once only. Prior to the study, local equine practitioners were informed of the project and requested to alert the investigators of any diarrhoeal episodes on the horse farms owned by their clients. In such instances, such farms are visited ahead of schedule to sample both on-going diarrhoeic episodes and non-diarrhoeic foals. 

### 2.2. Source of Horses

**Figure 1:**
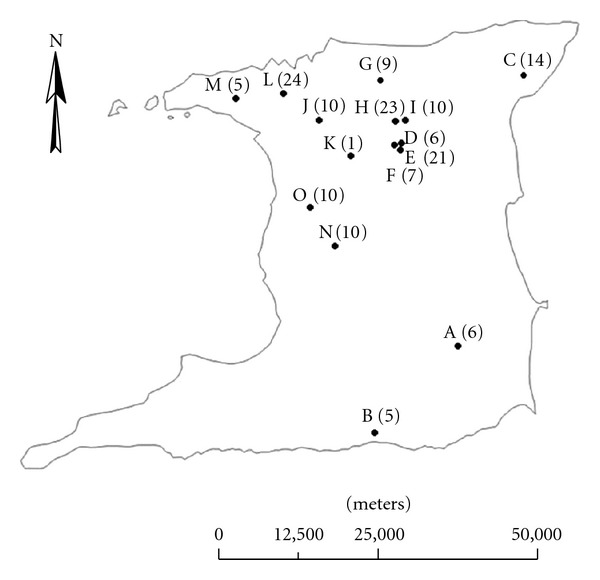
Map of Trinidad showing location of stud farms where foals (number of samples in bracket) were sampled for study.

Horse farms across the country identified through the assistance of the Trinidad and Tobago Racing Authority (TTRA), individual horse owners and practising equine practitioners. Overall, a total of 15 horse farms were identified and sampled across Trinidad as shown in Figure 1.

### 2.3. Case Definition

An animal was categorized as “diarrhoeic” if it had been experiencing bouts of watery diarrhoea for the 24 h or more prior to the farm visit. In most cases, faecal staining of the perianal area was observed.

### 2.4. Sample Size Determination

The sample size was determined using the formula = *z* ^2^ *xp*(1 − *p*)/*L* ^2^ [31], where *n* is the sample size, *z* is the confidence interval (1.96), *p* is the estimated prevalence = 17% [4], and *L* is the absolute error (2%). As the result of this formula was greater than the population size, it was therefore adjusted using the formula *n*_*a*_ = *n*/(1 + *n*/*N*) [31], where *n*_*a*_ is the adjusted sample size, *n* is the sample size as determined from the previous formula and *N* was 200, the total population of foals in the country during the period of this study. The sample size was hence determined to be 144. However, given the small number of foals in the population (164), it was decided to collect all available samples. For the study, the exclusion criteria were as follows: (a) foals older than 6 months of age; (b) animals undergoing antibiotic treatment at the time of sampling; (c) foals that have experienced recurrence of diarrhoea episodes less than 4 weeks after recovery following therapy prior to farm visit for sampling.

Overall, a total of 164 foals were sampled in the study.

### 2.5. Sample Collection

The foaling season in Trinidad spans from January to mid-May; therefore, sampling for this investigation was conducted from January 2010 to June 2010. A total of 15 Thoroughbred stud farms across the country were visited, and approximately 10 g or mL of faeces was obtained rectally from all foals and the samples identified. In most cases, gloved fingers were used to stimulate the anal area of diarrhoeic foals to stimulate defaecation and the faecal materials were collected into sterile faecal cups. A questionnaire, which elicited information on the age, sex, location of stud farms, management practices, experience of diarrheal episodes (past and current), and drugs used in the treatment of diarrhoea, was administered on each farm. The faecal samples (liquid, semisolid, and solid) were then transported to the laboratory chilled in tightly closed faecal cups which did not specifically create an anaerobic condition which may be suitable for *Clostridium* spp. All sample were processed upon arrival or within 24 h of collection whenever feasible. 

### 2.6. Detection of Enteric Pathogens

For the detection of *E. coli,* swabs of the faecal contents were plated onto MacConkey (MAC) agar and sorbitol MacConkey (SMAC) agar (Oxoid Ltd., Basingstoke, Hampshire, England) to detect *E. coli* and *E. coli* O157, respectively. Inoculated plates were incubated aerobically at 37°C for 24 h after which colonies that were reddish or pinkish on MAC agar were tentatively considered as *E. coli* and colourless or cream-appearing colonies on SMAC that were considered potential O157 serotype of *E. coli* were picked to inoculate blood agar plates. Inoculated blood agar plates were incubated overnight at 37°C, and pure cultures were subjected to biochemical tests for identification of *E. coli* using standard methods [32]. Colonies from blood agar plates which were representative nonsorbitol fermenting colonies of *E. coli* that originated from SMAC for each sample were subjected to the slide agglutination test by the use of *E. coli* O157 antiserum (Oxoid Ltd., Basingstoke, Hampshire, England).

 To isolate *Salmonella *spp., approximately 1 g of faecal materials was added to 9 mL each of selenite cysteine (SC) and tetrathionate (TT) broths for enrichment and incubated for 24 h at 42°C and 37°C, respectively. Enriched broths were subsequently subcultured onto xylose lysine desoxycholate agar (XLD) (Oxoid Ltd., Basingstoke, Hampshire, England) and brilliant green agar (BGA) and incubated aerobically at 37°C for 24 h. From each plate with growths, representative suspect colonies of *Salmonella *spp., which were pink colonies with black centres on XLD agar and pink colonies on BGA, were subjected to biochemical tests using standard methods [32]. Isolates biochemically identified as *Salmonella *spp. were subjected to slide agglutination tests using commercially available *Salmonella *polyvalent antisera A-I and Vi (Difco Laboratories Inc., Detroit, MI, USA). All isolates confirmed to be *Salmonella *spp. were sent to the Caribbean Epidemiology Centre (CAREC), Port of Spain, Trinidad and Tobago, the regional *Salmonella *typing laboratory, for confirmation and serotyping.

 For the qualitative isolation of *Clostridium *spp., faecal samples or faecal swabs were enriched in cooked meat medium (Difco Laboratories Inc., Detroit, Michigan, U.S.A.) by adding 5 mL of medium and incubated in an anaerobic jar at 37°C for 48 h. The growth was used to inoculate Shahidi-Ferguson Perfringens agar (Oxoid Ltd., Basingstoke, Hampshire, England) and incubated anaerobically at 37°C for 48 h. Suspect isolates were then Gram-stained. All isolates were identified as *Clostridium *spp. using standard methods [33, 34].

 The presence of rotavirus in faecal samples was detected by the use of a commercially available enzyme immunoassay, Rotascreen II EIA (Microgen Bioproduct Limited, Camberley, United Kingdom).

 To detect *Cryptosporidium *spp., direct smears of freshly collected faecal samples were made on glass slides and stained using the modified Ziehl-Neelsen staining procedure and identified as earlier described [35].

*Strongyloides westeri* was detected by faecal flotation in zinc sulphate followed by microscopic examination [35].

### 2.7. Determination of Resistance to Antimicrobial Agents

The resistance of isolates of *E. coli* and *Salmonella *spp. to antimicrobial agents was determined using the disc diffusion method. For the study, the following antimicrobial agents and concentrations were used: gentamicin (CN, 10 *μ*g), tetracycline (TE, 30 *μ*g), chloramphenicol (C, 30 *μ*g), sulphamethoxazole/trimethoprim (SXT, 23.25 *μ*g/1.75 *μ*g), streptomycin (S, 10 *μ*g) and ampicillin (AMP, 10 *μ*g). The breakpoints of the National Committee for Clinical Laboratory Standards, NCCLS [36], were used to determine the susceptibility or resistance of isolates to the antimicrobial agents.

### 2.8. Statistical Analyses

The frequency of detection of each organism was calculated and the data analysed using univariate assessment of the association between each organism and the occurrence of diarrhoea in the foals, location of farm, sex of foals, age of foal, and management practice on farms. The Chi-square test for independence was used to analyse each variable individually in their association with diarrhoea occurrence. The *P* value was set at alpha = 0.05 and 1 degree of freedom. The prevalence of resistance of isolates of *E. coli* and *Salmonella* spp. was related to the diarrhoeal status of foals to detect any statistically significant difference using the Chi-square test.

## 3. Results

### 3.1. Frequency of Detection of Enteropathogens in Faecal Samples

A total of 164 faecal samples were collected from foals during the 2010 foaling season; however, only 9 had on-going diarrhoea during the study period. The frequency was comparatively high for *Escherichia coli *(85.0%)*, Cryptosporidium *spp. (64.8%), and *Strongyloides westeri *(35.7%) as shown in Table 1. Relatively low frequency of detection was found for *Clostridium* spp. 12.9%, *Salmonella* spp. 4.4%, Rotavirus 2.1%, and *E. coli* O157(0.0%) as shown in Table 1. The following serotypes of *Salmonella* spp. were isolated: *Salmonella *Anatum, 3, 10 : e, h : 1, 6; *S*. Javiana 9,12 : 1, z28 : 1, 5; *S*. Uganda 3, 10, 1, z13 : 1, 5, and *S*. Aberdeen 11 : 1; 1, 2.

### 3.2. Frequency of Enteropathogens in Diarrhoeic and Non-Diarrhoeic Foals

The frequency of isolation of *Salmonella* spp. in diarrhoeic foals (33.3%), that is, 3 of 9 foals, was significantly (*P* < 0.05; *χ* ^2^) higher than found in non-diarrhoeic foals (2.7%), that is, 4 of 150 foals as shown in Table 2. *Salmonella* spp. of serovars *S*. Anatum (1 foal), *S*. Javiana (1 foal), and *S.*  Aberdeen (1 foal) were recovered from diarrhoeic foals compared with non-diarrhoeic foals which yielded serovars *S*. Anatum (2 foals), *S*. Uganda (1 foal) and *S*. Javiana (1 foal). Although *E. coli *was isolated at a higher rate (100.0%) in diarrhoeic foals compared with non-diarrhoeic foals (84.1%), the difference was not statistically significant (*P* < 0.05; *χ* ^2^). However, the frequency of detection of *Cryptosporidium *spp. and *Strongyloides westeri* was found to be higher in non-diarrhoeic than in diarrhoeic foals. For *Cryptosporidium* spp., the frequency of detection was 25.0% (2 of 8) in diarrhoeic and 67.2% (92 of 137) in non-diarrhoeic foals and the difference was statistically significant (*P* < 0.05; *χ* ^2^). For *S. westeri*, 20.0% (1 of 5) and 36.7% (29 of 79) of diarrhoeic and non-diarrhoeic foals, respectively, were positive for the parasite. The difference was however not statistically significant (*P* < 0.05; *χ* ^2^). 

Both *Clostridium* spp. and rotavirus were not detected in the faecal samples from diarrhoeic foals.

### 3.3. Frequency of Detection of Enteropathogens by Age of Foals 

**Figure 2:**
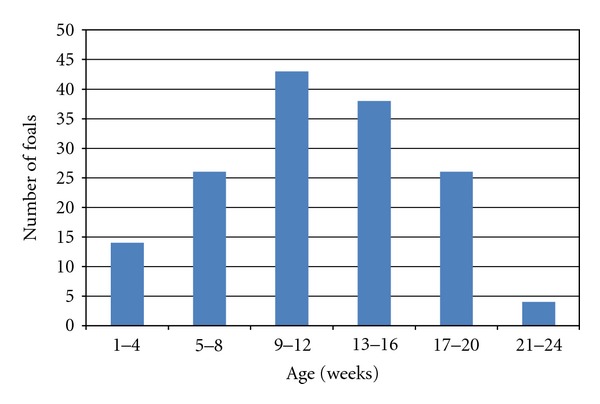
Age distribution of foals studied.

The age of the foal population was normally distributed with a range of 1–21 weeks and a mean age of 11 weeks (Figure 2). Overall, the ages of the foals could only be ascertained in 151 foals, due to the dearth of information as a result of inadequate records on the farms of new owners of these foals. 

**Figure 3:**
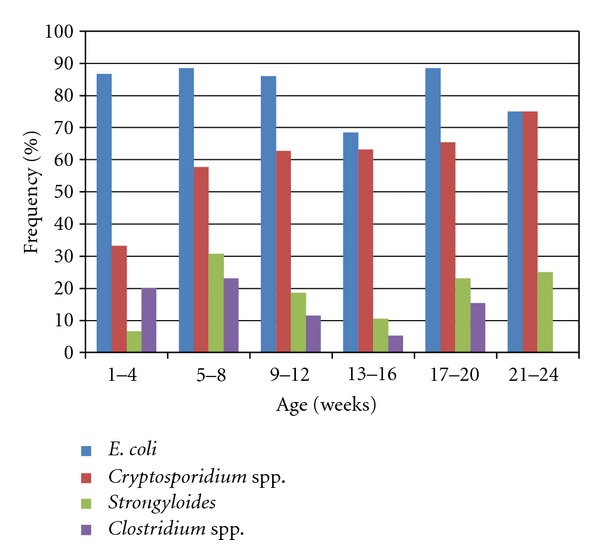
Frequency of detection of enteric pathogens in foals by age.

 Five (55.6%) of the 9 foals which had diarrhoea at the time of sample collection were approximately 1 month old while the remainder were approximately 4 months old. Six (85.7%) of the seven foals from which *Salmonella *spp. were isolated were aged 9–15 weeks old, while foals positive for rotavirus were in the 15-16-week old age bracket. For *Cryptosporidium *spp. and *S. westeri*, the age distribution of the foals positive for the microorganisms was similar to the distribution of the total foal population, while, for *Clostridium* spp. and *E. coli*, the peak frequency occurred in the age bracket of 13–16 and 5–8 weeks, respectively (Figure 3).

### 3.4. Frequency of Detection of Enteropathogens by Sex of Foals 

 For the 156 foals in the sample population whose sex was known, 65 (41.7%) were male while 91 (58.3%) were female. The sex of the remaining 8 foals could not be ascertained primarily because of movement of foals across farms and inadequate farm records on the foals. Diarrhoea was found to be more prevalent in male (6.2%) compared with female foals (4.4%), but the difference was not statistically significant (*P* > 0.05; *χ* ^2^). 

 The majority (80%), that is, 12 of the 15 stud farms had a higher number of female foals. *Salmonella *spp., *Cryptosporidium *spp., and *S. westeri* had higher frequency of detection in male (6.2%, 73.4%, and 51.4% resp.) than female (3.3%, 60.5%, and 25.5% resp.) foals. However, the differences were only statistically significantly (*P* < 0.05; *χ* ^2^) different for *S. westeri.* The frequency of detection of rotavirus infection was similar in male (1.7%) and female (2.4%) foals, while, for *C. perfringens* and *E. coli,* although detected at higher rates in female (15.1% and 80.8% resp.) than in male (10.9% and 80.0% resp.) foals, the differences were not statistically significant (*P* > 0.05; *χ* ^2^).

### 3.5. Farm Trends for Detection of Enteropathogens

*Cryptosporidium *spp. was detected on all the stud farms visited with a generally high farm prevalence ranging from 30.0% to 100.0%. *Salmonella* and *S. westeri* were detected from 5 (33.3%) each of 15 farms. A majority of farms, 75.0% (3 of 4), that had recorded cases of diarrhoea had *Salmonella-*positive foals compared with 18.2% (2 of 11) of farms without cases of diarrhoea. On one large farm, all the enteropathogens detected in the current study were found in foals. The frequency of detection of enteropathogens in foals per farms was not statistically significantly different across farms in the country (Figure 1). 

### 3.6. Practices on Stud Farms

Based on the questionnaire survey of the 15 stud farms, the following antimicrobial agents were used: penicillin-streptomycin (2 farms), furazone/furacin (2 farms), and gentamycin (1 farm). Based on the records most of the antimicrobial agents used on the farms were used without the prescription or supervision of veterinarians. It was also noted that there was a widespread movement of foals across stud farms in the country.

### 3.7. Prevalence of Resistance of *E. coli* and *Salmonella spp.* to Antimicrobial Agents

For the 6 antimicrobial agents tested, the frequency of resistance was higher for isolates recovered from diarrhoeic than from non-diarrhoeic foals (Table 2). The difference was however statistically significant (*P* < 0.05; *χ* ^2^) for tetracycline only, 85.7% (6 of 7) and 37.2% (45 of 121), respectively. For *E. coli* isolates from all sources, the frequency of resistance was relatively low to (12.5%) and chloramphenicol (1.6%) but high to ampicillin (89.1%), streptomycin (43.8%), and tetracycline (39.8%). 

 Two (28.6%) were resistant to tetracycline and ampicillin; and 1 (14.2%) isolate to gentamycin and chloramphenicol. All isolates of *Salmonella *spp. were however sensitive to sulphamethoxazole/trimethoprim and streptomycin. The differences in the frequency of resistance to antimicrobial agents amongst *Salmonella *isolates from diarrhoeic and non-diarrhoeic foals were not statistically significant (*P* > 0.05; *χ* ^2^).

## 4. Discussion

 It is of significance that all the enteropathogens tested for, with the exception of *E*. *coli* serotype O157, were detected in foals across stud farms in Trinidad albeit at varying frequency. All these enteropathogens (*E. coli*, *Salmonella* spp., *Clostridium* spp., rotavirus, *Cryptosporidium *spp., and *S. westeri*) have been documented to be causative agents of foal diarrhoea in several studies [2, 4–6]. The finding that these enteropathogens were detected in non-diarrhoeic foals which were predominantly (155 of 164 foals) available for the study is in agreement with published reports [6, 7, 23]. The fact that the current study is cross-sectional by design may have accounted, in part, for the findings. It is pertinent to mention that foals that have experienced recent episodes of diarrhoea prior to the farm visit but non-diarrhoeic at the time of sampling for the study may still be shedding the enteropathogen (s) responsible for the prior diarrhoeic episodes. Failure to detect statistically significant differences in the frequency of detection of enteropathogens between diarrhoeic and non-diarrhoeic foals as found in five of the six enteropathogens in the current study has been reported by others [6, 7, 23]. The effect of the low number of diarrhoeic foals available for the current study cannot also be ignored.

 The frequency of detection of *E. coli* (85.0%) was the highest of all the organisms assayed; however, this was not a surprise since the organism is part of normal flora of the intestinal tracts of animals [8] although it is also a known pathogen [9, 10]. In a study of Thoroughbred foals in Britain and Ireland, the prevalence of *E. coli* in diarrhoeic and non-diarrhoeic foals was similar [6]. The study did not however determine the occurrence of virulence markers or pathogenicity of the *E. coli* isolates from foals. In the current study, *E. coli* O157, known to be a verocytotoxigenic serotype, was not isolated from the 164 faecal samples tested. *E. coli* O157 serotype has however been recovered from diarrhoeic and non-diarrhoeic calves, lambs, and piglets across livestock farms in the country at a frequency ranging from 9.2% to 32.3% [30]. Horses, particularly foals, may therefore not be important reservoirs or carriers of this serotype of *E. coli* in Trinidad. *E. coli* O157, which is a cause of haemorrhagic diarrhoea in humans [37], has been reported by others to be isolated from the faeces of horses at a low frequency of 0.4% [38] but at a higher rate (12.3%) from horse farm environments [39].

 Based on the antibiograms of the *E. coli* isolates in the current study, it is evident that they were mostly sensitive to chloramphenicol and gentamycin but least susceptible to ampicillin and streptomycin. The detected resistance to both ampicillin and streptomycin could be explained, in part, by the fact that these antimicrobial agents were mentioned by the horse owners to be used at a high frequency on the stud farms. In a previous study conducted elsewhere on *E. coli* strains cultured from foals [9], the resistance was found to be highest to chloramphenicol and streptomycin and lowest to tetracycline and sulphamethoxazole-trimethoprim. Another finding in the current study with clinical relevance was the detection of statistically significantly higher frequency of resistance to tetracycline amongst isolates of *E. coli* recovered from diarrhoeic compared with non-diarrhoeic foals. This is an indication of a possible development of resistance due to misuse or overuse of the antibiotic in the treatment of foals locally. This has a potential to reduce the efficacy of tetracycline when used in the therapy of infections due to *E. coli* in foals in the local equine industry. It is therefore imperative that better monitoring and control of the use of antibiotics on the farms be practised. 

The frequency of isolation (33.3%) of *Salmonella* spp. from diarrhoeic foals in the current study is higher than the rates of 12% (28/233) [5] and 13% (60/465) [14] reported for diarrhoeic foals elsewhere but lower than the 35.1% (34/97) reported by Walker et al. [13]. The fact that the rate of isolation of *Salmonella* spp. was significantly higher than found in non-diarrhoeic foals suggests aetiological significance as earlier documented by others [4, 5, 11, 12]. A further indication of possible aetiological significance of *Salmonella* spp. is the fact that the *Salmonella *spp. were recovered from 83.3% of farms that had foals experiencing diarrhoea at the time of sampling. At variance with the findings in the current study, where there was a significantly higher rate of isolation of *Salmonella* spp. from diarrhoeic foals compared with non-diarrhoeic foals, are reports that the rates of detection between both groups of foals were similar [5, 13]. Adesiyun et al. [40] in a cross-sectional study of diarrhoeic and non-diarrhoeic livestock in Trinidad, similarly reported that there were no statistically significant differences in the isolation rates between animals that had diarrhoea 4.0% (21 of 523) and those that did not, 2.5% (8 of 324).

 In the current study, a majority of *Salmonella* spp. (6 of 7) were recovered from foals aged 2–4 months old, a finding in agreement with a published report which stated that the pathogen is more prevalent in foals aged 1 to 3 months old [41]. 

 The four serotypes of *Salmonella *spp. (S. Anatum, *S*. Javiana, S. Aberdeen and S. Uganda) representing the first reported documentation of these serovars from foals in the country. Other studies have reported the isolation of other serovars of* Salmonella* including S. Ohio [13], S. Typhimurium [15], S. Newport [14]. It is of interest to detect that in the current study, *S.* Anatum was the most frequently isolated serovar and represented 42.9% of the *Salmonella *isolates, a finding comparable to the report of Ernst et al. [14] where S. Newport and *S.* Anatum were the prevalent serovars isolated foals (diarrhoeic and non-diarrhoeic) at a frequency of 20% (12/60) and 13% (8/60) respectively. 

 The finding that all isolates of *Salmonella* spp. recovered in this study were sensitive to both sulphathoxazole/trimethoprim (SXT) and streptomycin is an indication that the two antimicrobial agents may be important in the chemotherapy of foal diarrhoea caused by *Salmonella* spp. Based on the questionnaire survey of the farmers during the study, the commonly used antimicrobial agents used to control foal diarrhoea are streptomycin and ampicillin.

However *C. perfringens* has been reported as an important causative agent for diarrhoea in foals some with fatal outcomes by others [3–6, 16]. In the current study, all the 9 diarrhoeic foals were negative for the organism compared with 13.4% of non-diarrheic foals being positive. The rather few number of diarrhoeic foals available for the study may also have been responsible for the 0.0% rate of isolation detected in this category of foals. In diarrhoeic foals, frequency of isolation of *C. perfringens* has been isolated with higher frequency, 18% (42/233) [5] and 57% (240/421) [4] elsewhere. The low frequency of isolation in the current study may be explained, in part, by the method of transportation of sample and isolation used. In the current study, faecal samples for diarrhoeic and non-diarrhoeic foals were transported in closed faecal cups which do not create an anaerobic condition for *Clostridium *spp. Also, it has been recommended by some authors that heat enrichment, not used in the current study, results in a higher isolation rate of *C. perfringens* compared with other methods [4]. 

 Rotavirus was detected at a very low prevalence (2.1%) being present in only 3 non-diarrhoeic foals approximately 4 months old, and all 8 diarrhoeic foals tested were negative for the pathogen. Rotavirus has been implicated in several outbreaks of infectious diarrhoea in foals which generally occurs as an epidemic with a high percentage of the herd being affected [3, 4, 17]. Adesiyun and Kaminjolo [30] reported that rotavirus was the only aetiological agent amongst enteric pathogens that was statistically scientifically associated with diarrhoea in livestock (calves, piglets and lambs) in the country. Again, the few number of diarrhoeic foals encountered in the current study may have made it difficult to assess the significance of rotavirus in foal diarrhoea in the country. It is, however, pertinent to mention that although rotavirus was only detected in non-diarrhoeic foals, this is considered the first documentation of rotavirus infection in foals in the country. It may therefore be necessary to further study the significance of rotavirus in foal diarrhoea in the country using a higher number of diarrhoeic foals. 

*Cryptosporidium parvum* has been reported to be a significant cause of foal diarrhoea in several studies [4–6, 21, 22]. In the current study, the rate of detection in diarrhoeic foals was 25%, which is higher than the 18% (12/67) reported for diarrhoeic foals in New Zealand [24] but considerably lower than a rate of 64% detected by Coleman et al. [22] in the USA. In our study, the overall frequency of detection of* Cryptosporidium *spp. in the foals (diarrhoeic and non-diarrhoeic) was 68.4% (94/145) which is much higher than reported by others which ranged from 7.4% (13/175) to 31% (9/29) [21, 23, 26, 42].

It was of interest to note that the pathogen was detected at a statistically significantly higher rate in non-diarrhoeic (67.2%) than in diarrhoeic foals (25.0%), an indication that *Cryptosporidium *spp. may not be an important causative agent of foal diarrhoea in the group of foals studied in the country, contrary to published reports. Again, the finding could be due to the few diarrhoeic foals available for the study and it is also relevant to mention that there are reports of similar isolation rates between diarrhoeic and non-diarrhoeic foals by others [6, 23].

 Endoparasites including *S. westeri*, have been associated with foal diarrhoea in several reports [5, 25] although it was reported that a significant association between the organism and diarrhoea was only detected when there were more than 2000 eggs per g of faeces [4]. The frequency of detection of *S. westeri* in diarrhoeic foals (20%) is considered high as is the overall frequency of 35.7% for diarrhoeic and non-diarrhoeic foals, compared to published reports which documented between 1.5% (11/733) and 6.0% (22/382) on stud farms in the USA. [27–29]. The rather high frequency of detection of *S. westeri* in foals sampled across stud farms in Trinidad reflects either absent or inadequate deworming programmes.

## 5. Conclusions 

 All organisms investigated were present in foals in Trinidad except *E. coli* O157, with *Cryptosporidium* spp.,* Clostridium perfringens *and *Strongyloides westeri* showing high frequencies of detection. Diarrhoeal cases were more common in foals 2–6 weeks old. *Salmonella* was statistically significantly associated with diarrhoea in foals in Trinidad and infection with this agent was most common in foals 2–4 months old. *Salmonella* spp. could therefore be considered important aetiological agents for foal diarrhoea in the country. Sulphamethoxazole-trimethoprim and streptomycin appeared to be the drug of choice for the therapy of salmonellosis in diarrhoeic foals. The high frequency of detection of *S. westeri* in foals sampled implies that deworming programmes in foals require more vigorous monitoring. Similarly, there is a need for a more prudent use of antimicrobial agents considering the finding of a high frequency of resistance to antimicrobial agents (ampicillin, streptomycin and tetracycline) amongst *E. coli* isolates recovered from both diarrhoeic and non-diarrhoeic). Sex appears to have played a part in infection with *S. westeri*, as there was a significant association between being male and being positive for *S. westeri*. Finally, the high frequency of detection of *E. coli*, *S. westeri*, *C. perfringens *and *Cryptosporidium *spp. in diarrhoeic and non-diarrhoeic foals coupled with the high frequency of resistance to antimicrobial agents amongst *Salmonella* spp. may have etiologic and therapeutic significance in foals in Trinidad.

## Figures and Tables

**Table 1:** Frequency of detection of enteropathogens in diarrhoeic and nondiarrhoeic foals.

Organism	All foals studied		Diarrhoeic foals		Nondiarrhoeic foals	
	Number of foals^a^ tested	Number (%) positive	Number of foals tested	Number (%) positive	Number of foals tested	Number (%) positive
*Escherichia coli*	160	136 (85.0)	9	9 (100.0)	151	127 (84.1)
*Escherichia coli* O157	158	0 (0.0)	9	0 (0.0)	146	0 (0.0)
*Salmonella* spp.	159	7 (4.4)^b^	9	3 (33.3)	150	4 (2.7)
*Clostridium* spp.	155	20 (12.9)	7	0 (0.0)	149	20 (13.4)
Rotavirus	145	3 (2.1)	8	0 (0.0)	141	3 (2.1)
*Cryptosporidium* spp.	145	94 (64.8)	8	2 (25.0)	137	92 (67.2)
*Strongyloides westeri*	84	30 (35.7)	5	1 (20.0)	79	29 (36.7)

^a^Comprising a total of a maximum of 9 diarrhoeic and 155 nondiarrhoeic foals.

^b^Four serovars of *Salmonella* spp. were recovered: *S*. Anatum (3 isolates), *S*. Javiana (2 isolates) *S*. Uganda (1 isolate), and *S*. Aberdeen (1 isolate).

**Table 2:** Frequency of resistance to antimicrobial agents amongst E. coli isolates.

Animal status	Number (%) of isolates resistant to the following						
	Number of isolates tested	CN	TE	C	SXT	S	A
Diarrhoeic foals	7	2 (28.6)	6 (85.7)	1 (14.3)	5 (71.4)	5 (71.4)	7 (100.0)
Nondiarrhoeic foals	121	14 (11.6)	45 (37.2)	1 (0.8)	36 (29.8)	51 (42.1)	107 (88.4)
Total	128	16 (12.5)	51 (39.8)	2 (1.6)	41 (32.0)	56 (43.8)	114 (89.1)

CN: gentamycin, TE: tetracycline, C: chloramphenicol, SXT: sulphamethoxazole/trimethoprim, S: streptomycin, and A: ampicillin.
